# Parental response to a letter reporting child overweight measured as part of a routine national programme in England: results from interviews with parents

**DOI:** 10.1186/s12889-016-3481-3

**Published:** 2016-08-20

**Authors:** Lawrence A. Nnyanzi, Carolyn D. Summerbell, Louisa Ells, Janet Shucksmith

**Affiliations:** 1 School of Health and Social Care, Teesside University, Middlesbrough, TS1 3BA UK; 2 School of Medicine, Pharmacy and Health, Durham University, Stockton on Tees, TS17 6BH UK

**Keywords:** Childhood obesity, Routine measurement programmes, Screening, Feedback, Parental views

## Abstract

**Background:**

Rising rates of childhood obesity have become a pressing issue in public health, threatening both the mental and physical well-being of children. Attempts to address this problem are multifaceted, and in England include the National Child Measurement Programme (NCMP) which assesses weight status in English primary school children in reception class (aged 4–5) and in year 6 (aged 10–11), with results being sent out to parents. However the effectiveness and impact of this routine parental feedback has yet to be fully understood. This paper reports one component of a mixed methods study undertaken in North East England, examining the impact of the feedback letters on parents’ understanding and feelings about their child’s weight status and whether or not this seemed likely to lead to behaviour change.

**Methods:**

One-to-one semi-structured interviews (*n* = 16) were conducted with a sample of parents/guardians after they had received their child’s weight results letter. Eight parents/guardians were sub-sampled from the group whose child had been indicated to be overweight or obese and eight were from the group whose child had been indicated to be of ideal weight status. Interviews were conducted until data saturation was reached for both groups.

**Results:**

The reactions of parents/guardians whose children were identified as being overweight followed a sequence of behaviours ranging from shock, disgust with the programme, through denial and self-blame to acceptance, worry and intention to seek help. On the other hand, the reaction of parents/guardians whose children were identified as being ideal weight ranged from relief, pleasure and happiness through affirmation and self-congratulation to ‘othering’.

**Conclusions:**

Whilst overweight and obesity is often portrayed as a medical condition, parents/guardians see it as deeply rooted in their social lives and not in health terms. Parents believe that the causes of overeating and lack of exercise relate closely to the obesogenic environment, particularly the complex social and cultural milieu and time pressures within which this sample of people live. Associating this problem in feedback letters with dangerous diseases like cancer, and advising parents to visit GPs to resolve child weight issues was perceived as inappropriate by the parents, and caused controversy and anger. Given the likelihood that the NCMP will continue as a monitoring device, it is evident that the management of the process needs to be reviewed, with particular attention being paid to the feedback process. Local health authorities will need to manage parental expectations and ensure linkage with appropriately commissioned remedial weight management interventions.

**Electronic supplementary material:**

The online version of this article (doi:10.1186/s12889-016-3481-3) contains supplementary material, which is available to authorized users.

## Background

The rising rate of obesity among children is one of the most pressing issues in public health [1–4]. Trends indicate that over the three decades between 1970 and 2000, there was a two to three fold increase in childhood obesity in many countries in Europe, Asia and the USA [5]. However recent studies indicate varying rates in different countries. In some high income countries rates are now stabilising and/or declining [6–8]. A review by Olds and colleagues [9] indicates that in Australia there have not been significant increases in the prevalence of childhood obesity in the previous 10 years. These findings are similar in other reviews conducted in the USA and France[10, 11, 12]. In countries which might be considered to be in 'nutrition transition', rates are rising steeply and the prevalence rates vary by socio economic status (SES) [12]. A growing body of research indicates that the obesity-related physical health consequences (e.g. cardiovascular diseases, insulin resistance, metabolic syndrome, type 2 diabetes mellitus) that affect obese and overweight adults are now known to affect obese and overweight children too [13–15]. Of particular concern is the fact that obese children are more likely to remain obese in adulthood, and childhood obesity has been shown to predict ill health later in adult life [16].

In light of the known consequences of childhood obesity, the UK government in 2004, as part of its Public Service Agreement (PSA) targets, identified the need to halt the year on year rise in obesity among children aged below 11 years in England by the year 2010 [1, 17]. In order to monitor the progress of the PSA target, the UK government in 2006 introduced the National Child Measurement Programme (NCMP) in which the height and weight of children would be measured by school nurses as children entered primary school in the reception year (aged 4–5) and as they left in year 6 (aged 10–11). The NCMP was established to inform local planning and service delivery, and to ensure appropriate targeting of weight management resources [18]. Height and weight figures for children are transformed into a Body Mass Index (BMI) score. BMI is a proxy for body fatness, and standard tables and graphs of population norms for BMI exist, against which the values for an individual child can be compared. Using standard cut-off points, BMI is used to derive categories of weight status in children.

In England, the UK90 [19] growth reference is used to calculate age and sex appropriate cut-off points for weight status, whereby, for population monitoring and surveillance purposes the following categories are used: obese (≥95^th^ percentile); overweight (≥85^th^ and < 95^th^ percentiles); healthy weight (>2nd and < 85^th^ percentiles); and underweight (≤2^th^ percentile) [20].

Although originally the NCMP was intended for general population surveillance purposes, it was subsequently manoeuvred towards becoming a screening programme, following the introduction of routine parental feedback. The original design of the programme did not involve identifying ‘caseness’, as it was originally intended as a population level surveillance programme. Only those parents/guardians who were particularly interested in knowing the BMI results of their children and who made contact with the Primary Care Trust (PCT)^1^ – a form of local health authority whose functions have now transferred to English and Welsh local authorities – would actually receive them [17].

However, soon after its implementation the programme received a great deal of attention from health professionals, researchers, politicians and the media. It was believed that routine feedback to parents could provide an opportunity to raise awareness of unhealthy body weight amongst families [17, 21]. Consequently in 2007, the Department of Health came under pressure from a House of Commons Select Committee to announce changes to the NCMP and in 2008/2009 it was announced that it would become a requirement for PCTs to deliver universal feedback of height and weight measurements to parents and their children, provided the resources were available [22]. With the introduction of this new recommendation the programme essentially changed focus from a surveillance programme to a screening programme.

Routine feedback of weight results to parents might potentially be of great importance since a growing body of evidence points to the fact that many parents are unable to identify their overweight children as being overweight [23–27], possibly due to the normalisation of overweight status in many communities. Routine feedback of BMI results has been practised over a much longer period in a number of states in the USA [28, 29]. However this strategy for combating obesity has been heavily criticised for overemphasising personal responsibility for obesity rather than promoting a more contextualised approach that focuses on the role of the environment and society in promoting obesity [30].

Overall, the NCMP is now quite well accepted and embedded within routine practice. However, critics have voiced the fear that a measure taken to promote better physical health in children may inadvertently result in poorer mental health, with both parents and children feeling stigmatised and anxious about the child’s weight status [31]. Aphramor [32] has discussed the stigmatising potential for such activity and has posited an impact on children’s self-esteem, but there have been few studies which followed children and parents/guardians through the measurement and feedback process to see if this is indeed an outcome that can be expected in a majority or minority of cases in the UK context.

The body of evidence examining whether parents/guardians either like or benefit from receiving the height and weight measurements of their children from school-based measurement programmes is equivocal. Nihiser and colleagues [29] reported that many studies in the USA indicated that parents prefer to be sent the results indicating whether or not their child was overweight provided they are expressed in a neutral way without placing any blame on anyone. However the UK and the USA have different health and education systems, so it is important to try to establish data derived in a UK context. A study conducted in England by Grimmett and colleagues [33] was amongst the first to try to establish the attitudes of UK parents to weight feedback on their children. In their study 50 % of the parents with overweight children reported changing health behaviour as a result of the weight feedback they had received. However this was an experimental study set up with parents who had opted to be involved and were thus arguably less than representative of the population as a whole and perhaps particularly of that fragment of the population whose children were most likely to be overweight or obese, and it was undertaken before routine feedback of height and weight measurements from local health authorities had been implemented nationally.

More recent studies have reported the potential for weight feedback to improve the ability of parents to recognise weight problems in their children. Falconer and colleagues [34] published results of a survey conducted in a sample of 1844 parents with children aged 4–5 and 10–11 years old before and after receiving weight feedback as part of the 2010–2011 NCMP cycle. They reported that the percentage of parents who could accurately recognise overweight in their child following weight feedback increased from 21.9 to 37.7 %. These results seem to have high external validity owing to the large sample size studied, but tell us only about improvements in recognition of the problem and nothing about consequences of that improved recognition. In another recently published qualitative study Syrad and colleagues [35] interviewed a sample of 52 parents who had received weight feedback as part of the 2010–2011 NCMP cycle. Results indicate that parents do re-evaluate their child’s weight status after receiving weight feedback, but the study does not look at change over time in parental response and one is thus led to conclude that the decision to act on the knowledge received is simple and instantaneous. These studies highlight the potential benefit of weight feedback in tackling childhood obesity, but it is clear that UK local authority public health teams and those with an academic interest in the issue need UK based evidence that adds to the survey data indicating that feedback methods are potentially effective, by looking more deeply at the mechanisms by which parents move towards acceptance of a problem and start to search for a solution. This study presents data which makes a new contribution in this area, and specifically looks at differences across the parent group and brings in a consideration of time as a fourth dimension which has often been seriously neglected in other studies.

## Methods

The qualitative study reported here involved individual interviews, and was part of a larger study which used a sequential mixed methods study design with a survey preceding the qualitative study [36]. The study was conducted in a single health authority in the North East of England, which has a relatively high prevalence of childhood obesity compared with other areas of England [1]. The basic sampling unit were primary schools in the health authority catchment area. The sampling frame was the list of all primary schools in the health authority. It was estimated that 24 schools would be required to obtain the required sample size of children. Participating schools were selected using a proportionate stratified random sampling technique. Schools were classified according to deprivation status using percentage of children on free school meals in a school as a surrogate measure of poverty. Of the 66 eligible schools, 39 were categorised as highly deprived, 12 as moderately deprived and 15 as low deprived. From the highly deprived category 14 schools were selected, four schools from the moderately deprived category and six schools from the low deprived category. Selection was done using tables of random numbers. Once a school was selected, all children aged 10 years and 11 years were approached and asked to take part in the study. If a school declined to take part, it was replaced using a similar procedure described above. Prior to measurement, 500 eligible children were approached for consent to take part in the questionnaire survey. Children were included in the study only if their parents/guardians had consented to them being measured in the NCMP and were excluded if they had physical disabilities or learning difficulties in accordance with the NCMP exclusion criteria. Out of these 267 were eventually recruited into the survey with full consent. Out of this number 264 children (53 %) completed the questionnaire. Later, at some point in the academic year 2009–2010, children participated in the NCMP where they were weighed and measured by the school nurse and parents/guardians subsequently received their child’s weight status measurements (feedback) from the health authority. Soon after this children were given a pack containing the demographic data collection form to take home to their parents. On this form, parents were asked to write the weight and height measurements they had received as feedback from the health authority. This form involved questions on age, ethnicity, education, and occupation of the parents/guardians, as well as the height and weight figures received as feedback from the PCT. 115 children returned the demographic data collection form with the section for the NCMP results completed. At this point all 115 children and their parents/guardians were asked if they would be prepared to take part in a follow up interview study.

**Fig. 1:**
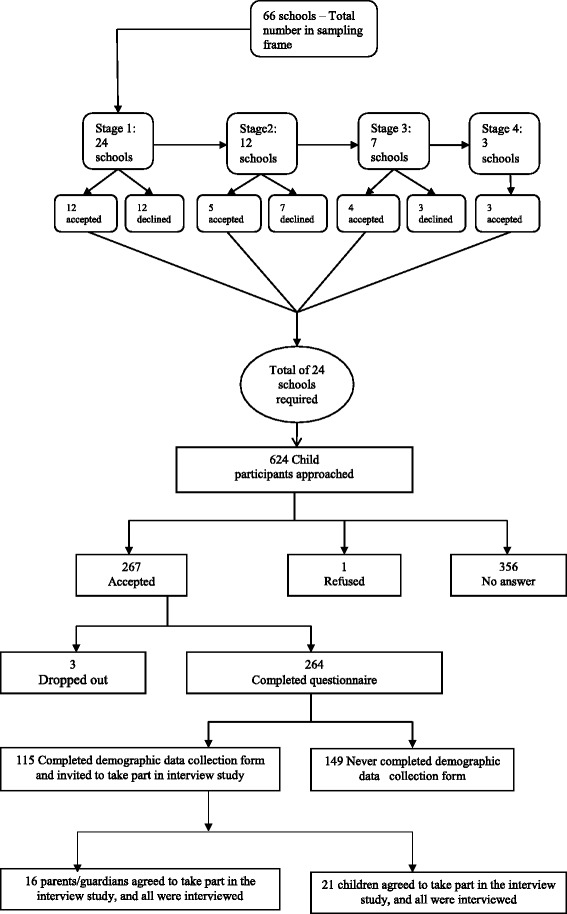
Procedure for selecting study participants. Shows the procedure for selecting study participants. Participants for the survey questionnaire were selected first then participants for the qualitative interviews were selected next

The parents/guardians who were selected for the qualitative study, which is the focus of this paper, were approached by letter through their child’s school. Twenty one parents/guardians agreed that their child could be interviewed and 16 gave their own consent to be interviewed also. All 16 adults who volunteered to take part were interviewed. Parents/guardians were asked to nominate where they would like the interview to take place, and the most convenient time, as well as their contact telephone number. Although this was essentially therefore a volunteer sample, saturation was reached in the data at an early stage, but all interviews were completed. Of the parents/guardians in the interview group, eight had a child who was indicated to be either overweight or obese by the NCMP feedback and eight had a child who was indicated to be healthy weight. The interviews took place between March and July 2010, and were conducted by the first author. Figure 1 shows a flow chart for the recruitment procedure.

Data from parents/guardians were collected using one-to-one semi-structured interviews. Interviews were carried out in homes for some participants or in a neutral location to suit the interviewee for others, with each interview taking approximately 60 min. Topics explored in the parent/guardian interviews included awareness of the NCMP, experiences before and after child participation in the NCMP, experiences before, during and after the feedback process. The full interview schedule can be accessed from Additional file 1. All the interviews were audio recorded to ensure that vital information was not lost or missed out in the analysis.

Interviews were transcribed verbatim and the transcripts were analysed using the thematic content analysis method following the method established by Burnard [37]. This method was chosen because it allows the researcher to immense him/herself into the data, which enables identification and proper interpretation of the themes rooted in the data [38]. Coding of the data was done by two researchers (LN and JS) independently to generate themes and categories. The independent lists were later merged to generate a comprehensive list of the themes and categories which covered all the accounts of the respondents. Audit trails were kept to increase the credibility of the findings. Ethical approval to conduct the study was obtained from Teesside University School of Health and Social Care Research Ethics and Governance Committee and consent from participants was obtained through signed consent forms on the day of the interview.

## Results

The majority of parents/guardians taking part in interviews were female (*n* = 13). Most parents/guardians (*n* = 8) could be categorised as having a low SES (when percentage free school meals in their child’s school is taken as the proxy measure for SES). Out of the 16 parents/guardians, eight had received a letter from the PCT indicating that their child was either overweight or obese; eight had received a letter indicating that their child was a healthy weight. A sample feedback letter can be accessed from the Department of Health archives [39].

This paper reports only one of the seven major themes that emerged from the post measurement interview data, namely the cycle over time of emotional reaction in families following receipt of the feedback letter. The other themes (not reported here) were challenges of parenting in contemporary society, the ‘obesogenic environment’ - a challenge for childhood obesity control, accessing help within the health system subsequent to weight feedback from the measurement process, enhancing awareness of weight problems for behavioural change, impact of the measurement process on mental wellbeing of children, challenges of the measurement programme [36].

### Reaction amongst parents/guardians of ideal weight children

**Fig. 2:**
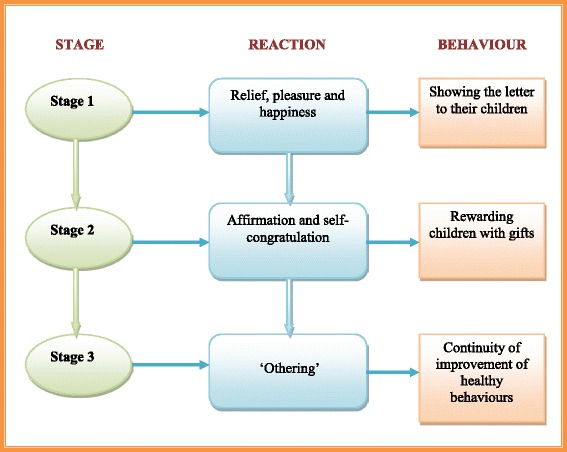
Sequence of events following receipt of child feedback for ideal weight children. Shows the sequence of events that parents/guardians whose children were indicated to have the ideal weight status followed after receiving the child weight feedback letter

The accounts of parents/guardians whose children were indicated to have the ideal weight status followed a sequence of events summarised in Fig. 2. This process is characterised by three main stages, each demonstrating distinct reactions and behaviours. In the first stage parents/guardians reported feelings of relief, pleasure and happiness soon after receiving the weight feedback letter. For instance, a parent remarked:

……I was quite happy that he was in the normal brackets (parent 14 – child of ideal weight).

However, it was evident in the interviews that parents/guardians had been unsure whether or not their children **would** be the correct weight before receiving the feedback letter. A number of parents/guardians had perceived their child as underweight.

….. I always thought, ‘Oh! Maybe she is underweight’, you know, but she wasn’t. So when I first read it, I went, ‘Oh! This is fantastic.’….. (parent 12 – child of ideal weight).

A small number of parents/guardians (*n* = 3) perceived their children to be the correct weight for their age and height, so the feedback letter confirmed what they had suspected all along.

….. It just confirmed what I always suspected anyway, so it was okay…. (parent 03 – child of ideal weight)

Parents/guardians then entered a second stage characterised by affirmation and self-congratulation. Feelings of having done their duty as good parents were evident in the accounts. We know from the children’s interviews [not otherwise reported here] that some were rewarded with parties and gifts as if they had succeeded in an exam. Parents/guardians subsequently appear to have entered into a third stage characterised by ‘othering’ – a process that identifies those that are thought to be different from oneself or the mainstream [40]. In this stage, parents/guardians saw themselves as part of the group that is doing the right thing, and viewed other people, especially those whose children were indicated to have weight problems, as not doing things correctly. Parents/guardians held the view that those parents/guardians whose children were obese/overweight needed to do something about it, rather than just blaming the health authorities for informing them about the weight status of their children. For instance a parent said:

…….I mean it’s not too late, they can do something about it. What’s the point of their saying, ‘I don’t need the bloody government telling me my child is overweight. They can’t even run the country’. People with obese children become really defensive instead of becoming aware of it. I would guess, and I am not being funny, these parents probably know their kids are overweight. If you know your kid has been portly and is being classed as being obese or whatever, you need to probably think and know, yeah, that can’t be wrong…. (parent 03 - child of ideal weight).

Some parents/guardians (*n* = 2) however recognised this reaction from parents of overweight children as being an instinctive protective behaviour on behalf of their children. A parent commented:

..... parents are just protective aren’t they? It’s a mother’s instinct to protect your children. Obviously you are not sort of adding a few pounds just to try and annoy the mother are you? It all starts with them putting things in their hands, in their mouth isn’t it? Instead of trying to say, ‘My God I have to do something about it’, they are not. They are saying, This is wrong, the scales are wrong, aren’t they? (parent 12 - child of ideal weight).

Similarly, some parents/guardians (*n* = 4) felt that other parents with overweight and obese children were responsible for making their children gain weight. A parent said:

……they are the ones feeding the kids and if they (the children) are overweight they are not feeling good about themselves….. The letter just looks to enforce that. Not only you are overweight, but the kid is overweight as well; and it’s your fault….. How is that not your fault really? (parent 13 - child of ideal weight).

Following affirmation of the child being the ideal weight for height, parents indicated that the feedback letter gave them reassurance to continue giving their children healthy foods and encouraging them to do more physical exercise. For example, a parent noted:

……It means I am feeding my children in the right way and I will continue doing just that…I will also encourage them to continue exercising….. (parent 07 - child of ideal weight).

### Reaction amongst parents/guardians of children indicated to have weight problems

**Fig. 3:**
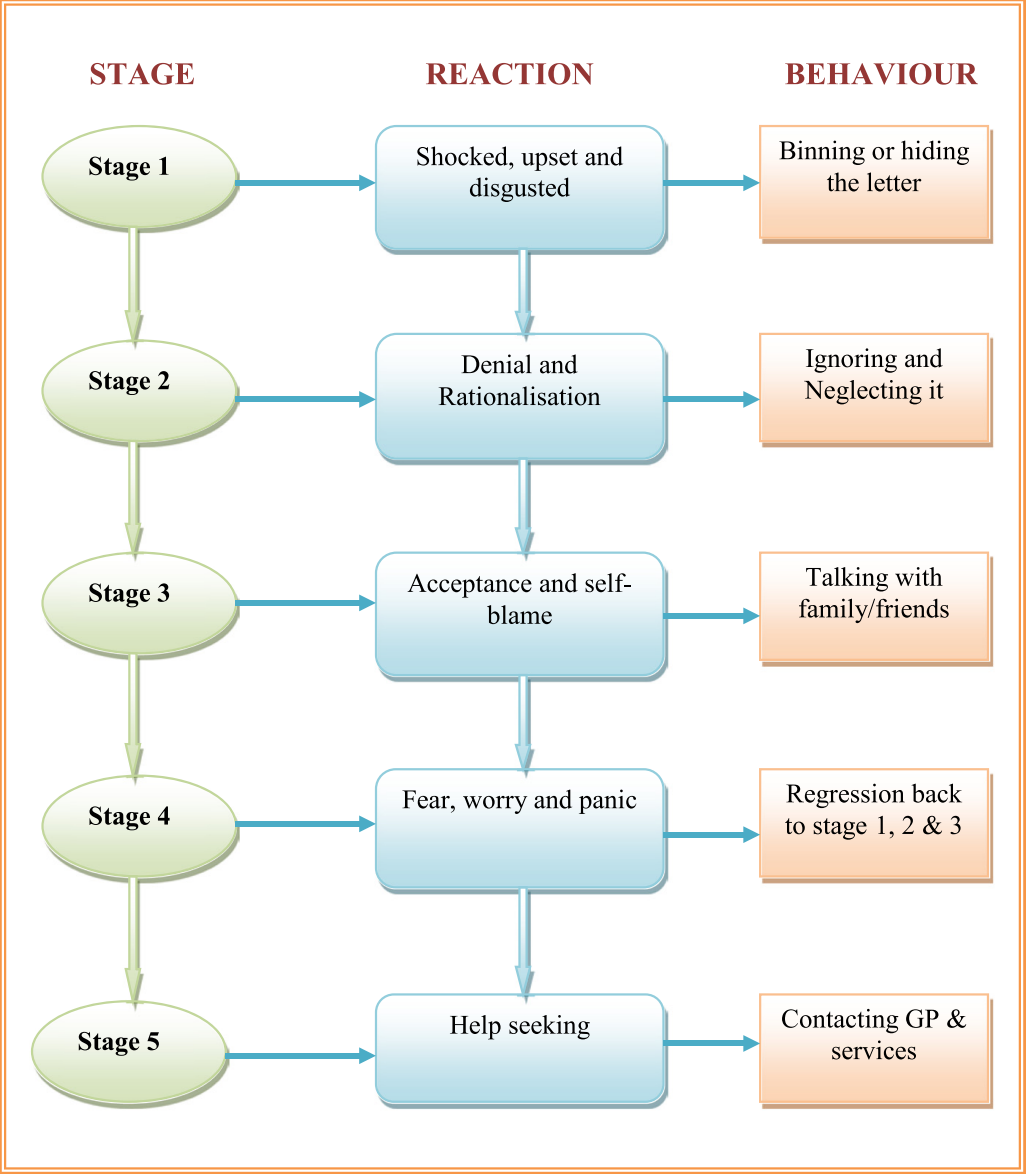
Sequence of events following receipt of child feedback in overweight children. Summarises the sequence of events that parents/guardians whose children were indicated to be overweight/obese followed after receiving the child weight feedback letter

Generally parents/guardians whose children had been labelled overweight/obese were upset about the news. These parents/guardians reported that it had never occurred to them that anyone would regard their children as overweight/obese and felt that it added to the ‘insult’ to see words like ‘overweight’ and ‘obese’ in bold letters. It is quite evident that this feedback caused a lot of panic and worry among families, as they were caught unawares. Parents/guardians reacted differently to the news. Some parents/guardians (*n* = 3) chose to throw away the letter, determined not to let their children see it, as they feared that this could impact on their children’s self-esteem and mental wellbeing. Others (*n* = 2) chose to sit their children down and tell them the news, but in a tone that did not create panic. The sequence of events that occurred from the time of receipt of the feedback seems to represent a process characterised by a similar pattern of parental behaviour in different families. This process has been summarised in Fig. 3.

This process comprises of five stages, each characterised by a particular set of reactions and behaviour pattern that parents/guardians demonstrated from the time they received the weight feedback onwards. Soon after receiving the feedback describing children as being overweight or obese most parents/guardians (*n* = 7) reported being ‘absolutely’ - ‘shocked’, ‘horrified’ and ‘disgusted.’ As a result these parents/guardians opted either to throw the letter away in the bin or to hide it so that their children could not see it. A mother whose child had been labelled overweight/obese told of her initial reaction:

….my initial reaction was, I was really shocked at the content of the letter. I was horrified, absolutely horrified, because out of the whole family you see I am sort of quite plump. My youngest daughter is probably a little bit overweight, but DJ is the one I would say, under no circumstances she is overweight (parent 01 - child obese/overweight).

Another parent, when asked whether or not she had discussed the letter with her son, said:

.......I was so shocked when I opened the letter and I read the contents because I always thought that my son was the right weight. Honestly, I have had no concerns at all about his weight or about what he eats or anything, so I didn’t even tell him we got it. I hid it away, because I don’t want to encourage him to be so conscious of his weight and I think he has the right attitude towards food and exercise (parent 10 - child obese/overweight).

While this mother just hid the feedback letter, some other parents/guardians (*n* = 3) decided either to destroy the letter completely or to throw it away in the bin. A mother whose son had been labelled overweight told of her disgust.

......all along I thought my son was absolutely fine and then it had on the letter that he was very overweight and the certain illnesses that he could get when he is older which I was quite horrified about, and then I put it in the bin so he couldn’t see it cos I didn’t wanna worry him. Obviously he is old enough to read. You know what I mean? (Parent 02 – child obese/overweight).

It was quite evident that, after the initial shocked reaction, most parents/guardians whose children had been labelled overweight/obese entered a stage of denial of the judgement. Parents/guardians (especially mothers) reviewed their child’s weight status, eating habits and physical exercise and found no problem. Most (*n* = 5) spoke of their children leading a very active lifestyle, eating a balanced diet, doing a lot of outdoor activities and it therefore being incredible that anyone could label their children overweight/obese. During this stage parents/guardians exhibited a range of behaviours. Some parents/guardians (*n* = 3) chose to ignore and neglect the feedback letter completely; others just wanted to sweep it under the carpet suggesting that it was not an issue for their children. For instance a mother said:

.....I really thought at the time that she was at a very healthy sort of weight range. She is sort of within what I believe she should be. She does lead a very active lifestyle, she dances and she does sort of quite a lot of outdoor activities and things and, like I said, she does eat a very healthy balanced diet. I would like to sort of just brush it under the carpet (parent 11 – child obese/overweight).

But some parents/guardians (*n* = 2) could not just let the feedback go without some response, so they took it upon themselves to write back to the authorities describing their disgust about the letter they had received. One parent, mistakenly assuming the research team to be culpable as far as the feedback letter was concerned, wrote to the researchers as follows:

To whom it may concern,

I would like to express my disgust after receiving a letter from the school nursing team stating that my daughter is overweight. At 11 years of age she is becoming increasingly aware of her image and is self-conscious about the way she looks. Thankfully she did not see the letter we received stating in bold lettering that she was supposedly overweight. Had she done so, I think it would have been a real blow to her confidence, possibly with detrimental effects to her health, as she may decide to change her very healthy attitude to eating and exercise? I think letters such as this can be as damaging as they can be helpful. I have no concerns about my daughter’s weight and believe that she is sufficiently active to maintain a healthy weight.

Yours sincerely,

Parent 01 – child obese/overweight.

This parent, after speaking with the researcher in the interview, reported feeling much better that she had spoken to someone about it. This underscores the importance of following up the weight feedback to try and provide the opportunity for parents/guardians to vent their anger. This mother said:

…..I am glad that I have had the opportunity to speak to you about it, because at the time I did feel very strongly about it, but maybe there were some other parents that felt as strongly but just didn’t do anything about it afterwards and just sort of went, ‘Oh well’ (parent 01 – child obese/overweight).

It was clear from the interviews that parents/guardians did not know who was responsible for the weighing and feedback process and therefore who to contact subsequently.

But sooner or later most parents/guardians who had denied their child’s weight status started to come to terms with it, recognising that denial would not solve anything. They reported reviewing their behaviour over time and realising that probably they hadn’t been feeding their children healthily. At this point they were entering into an acceptance stage. Many parents/guardians in this stage reported relying on friends and family, so they discussed the feedback with either their own parents, friends or neighbours to try and examine what might have gone wrong. In this stage parents/guardians fully recognise that their children could be overweight. A parent of an overweight child said:

.....I think I studied it for a while and I just thought about it for a while and I was just a bit sort of shocked and I kept thinking, ‘Well, is she?’ I sort of discussed it with my mother and things like that and sort of discussed it with a few people and the more I thought about it I thought: Well yes, I agreed for her to be in this study and I do have to agree with the letter. Maybe she is actually overweight (parent 01 – child obese/overweight).

Eventually many parents/guardians (*n* = 7) of overweight children became worried and started to worry over what they should do. In the current study, some parents/guardians reported feelings of disappointment and anger similar to the reactions in stage 1. But also it is at this point that parents/guardians wanted some answers about what they could be doing wrong and this led in some cases to an attempt to seek help. Some parents/guardians (*n* = 2) at this stage phoned the numbers given on the feedback letter to try and find out what could be done differently in order to fix the weight problems of their children. For instance some parents/guardians reported:

.......I didn’t think he was 9 stone 7lbs, but he was. In fact I was worried, but I knew it wasn’t the end for me. It was obviously then some of the other mothers were talking about things like that, and I know a few other mothers were concerned and then also got on the phone...... (parent 02 – child obese/overweight).

In reviewing the ways in which parents respond to the NCMP feedback letter it is therefore important to distinguish the reactions of those deemed normal or ideal weight and those deemed to be overweight or obese, as reactions are quite different. For both groups, there is a process of adjustment to the news. In those whose children are deemed to be ideal weight, relief and self-congratulation are followed by a somewhat unhelpful 'othering' process, which pays scant regard to the fact that many were unsure of their own child’s weight status prior to the arrival of the letter. In those for whom the feedback letter brings a judgement of overweight/obese on their children, shock is quickly followed by a range of denial behaviours (some passive; some very angry). Eventually some will engage in lifestyle review and a small number will go on to seek help to remedy the problem identified.

## Discussion

Exploring the entire process over time that parents/guardians go through when they receive weight feedback for their children and how this differs across the parent group could provide an important step in identifying the best timing for interventions and how best to follow up the weight feedback letter in families where children are indicated to have weight problems. In reviewing the literature, few other studies that explore the emotional process of receiving and making sense of weight feedback were found. Local arrangements for delivering weight feedback are very varied, with no overarching review at present of the relative success of these hundreds of ‘natural experiments’. Thus authorities differ in the length of time it takes them to feedback, whether they accompany the letter with additional written information, whether they follow up the letter with a telephone call in those cases where children are perceived as having the most severe need for intervention, and so on. The results of this small study indicate a much greater need for attention to these process details if behaviour change to reduce lifestyle behaviours associated with obesity is the end goal. In particular the point of follow up intervention may be critical if the cycling of emotions evident in this study is typical.

This study adds to the findings from a number of other recent studies which have examined parental reactions. Mooney et al [41], in a postal survey followed up by telephone interviews, also found shock to be the principal first reaction of many parents of overweight children, most of whom had not previously considered their child to be overweight. As in this study, reflection had persuaded many that they should do something, but it was unclear whether many had followed this through with changes to lifestyle or knew where to go to seek help and support. The current study notes that ‘shock tactics’ (such as associating the child’s current overweight status with very adverse health outcomes like cancer in later life) are rarely effective when the danger forecast is not immediate, and may in fact mitigate against help-seeking behaviour.

Syrad and colleagues [35] reporting on interviews with parents of children deemed overweight in the NCMP exercise, noted parents refuting the judgement made in the ‘fat letter’ on the basis that it acknowledged none of the variations in build, disposition and inherited factors of their children and that the linkage of obesity with health belied the evidence of their own eyes in terms of children’s happiness and wellbeing. In a study undertaken in PCTs in and around London, where the authors acknowledge that their recruitment methods may have produced a sample which was a largely volunteer one and which was therefore possibly more ‘invested’ and better educated than our own, parents were confidently using their own judgement to temper the verdict given to them in the feedback letter and were thus less likely to use it as a spur to action.

A number of reasons might explain the emotional reactions families go through when they receive weight feedback. First and foremost, society too often associates being overweight/obese as being equivalent to being stupid, lazy and unable to control oneself. Hill and Silver [42] discussed the negative attributes society holds towards overweight and obesity. Therefore parents are unwilling to consider their children to be overweight because they reject the associated negative attributes. Ultimately this could explain why the news of the child being overweight/obese is upsetting, disgusting and annoying. Boutelle and colleagues [43] also discussed the unwillingness of parents to consider their children obese or overweight even when they recognised it in their children. But there could also be a genuine inability among parents/guardians to distinguish overweight/obesity from normal weight among children. This could be central to the observation made in the current study that parents/guardians receive the news of their children being overweight/obese with utter surprise, as they genuinely previously thought that their children were ideal weight.

The current study identified further factors that instigated unnecessary panic and annoyance among parents/guardians. It became clear that sending the weight feedback letter with words such as overweight/obese in **bold lettering** only added to the insult of labelling children overweight/obese in the view of parents/guardians. Highlighting such terms is interpreted as judging parents and placing blame on them. It individualises blame and shifts the entire problem of childhood obesity from the wider cultural and social perspective, as highlighted in the Foresight Report [30], stigmatising individuals and their parenting practices. In this study the rapid cycling in the emotions of the parents of the ‘ideal weight’ children from relief to ‘othering’ and stigmatising the behaviours of parents whose children had not passed muster illustrates perfectly how easy this transition is made by the NCMP exercise.

This study also identified that implicitly linking obesity to fatal illnesses in the feedback letter is one of the factors that created unnecessary panic and annoyance among families. In the first place it is arguably morally wrong to tell a child of 10–11 years that they are more likely to die of **cancer**, diabetes, cardiovascular diseases etc. if they do not stop putting on weight. Apart from creating unnecessary panic among families, it serves to medicalise the whole issue of child weight status. It is perhaps not surprising that the PCTs recommended that parents/guardians visit General Practitioner (GP) surgeries and school nurses, but it could be argued that it is disingenuous, given the lack of preparedness of the one service and the under resourced nature of the other [44]. It ought to be understood that, from the parents’/guardians’ point of view, child weight status is not a medical problem; parents/guardians do not see it as necessitating visiting GP surgeries [35]. This disconnection in the way of thinking about child weight status between the health authorities running the NCMP and the parents/guardians is only likely to lead to conflicts and negativity regarding the interventions aimed at combating child weight problems. Consequently the child weight feedback letter may end up engendering feelings of blame amongst the individual families and this could be counterproductive in achieving the wider public health goals intended by implementing the programme. The weight feedback letter could be a very important factor acting as a spur to families to think of adopting healthy lifestyles. Inducing families into a state of readiness to change is pivotal in any public health promotion interventions [45]. If the weight feedback letter is one of the factors that could induce families into the state of readiness to change then it should be supported. Notwithstanding this, there is a need to take special care to ensure that the message is put across sensitively and in a correct way to minimise as much as possible any panic and annoyance among families which could create undesirable outcomes. Public Health England currently recommends avoiding use of such terms as obese in the weight feedback letter and to stick to a time frame for sending out the feedback.

One of the important aims of the current study was to identify what families do when they receive child weight feedback. It clearly identified the unwillingness of parents/guardians to visit medical professionals about their child’s weight status, and willingness to change health behaviour among families seemed to be strongly hampered by the lack of knowledge of the system and how to find help within it. Most families found the NCMP process too complicated. It was clear that they did not know where to go for help and who to contact. Although the PCT letter indicated to them where they should go for help, namely – their family doctor; it seems the PCT may have been pointing families in the wrong direction. While health authorities inevitably medicalise the issue of child weight status, families do not view child weight status as a medical problem. Child weight status is viewed by families as a social problem, and this explains why almost all parents did not consider seeing the GP about their child’s weight status.

School – based weight monitoring programmes such as the NCMP are part of the wider public health agenda to promote better health and wellbeing. However results from this study highlight wider public health issues regarding the right timing for interventions following weight feedback. Health authorities have been sending additional materials with the weight feedback letter suggesting to parents where they could go for help; such information is likely to have been discarded along with the original letter. The findings from the current study have shown that most parents did not even see these materials and many confessed that these materials ended up in the bin. Apart from wasting resources, this sort of practice does not achieve any of the public health goals intended. A reasonable approach to tackle this issue could be to send these materials separately as a follow up package, some time after the feedback letter has been sent to the homes of children indicated to have weight problems, and also some time after initial hostile reactions to the feedback have abated somewhat.

### Implications for public health policy and practice

There are a number of aspects of the current NCMP feedback process which should be reviewed, which could reduce unnecessary panic, worry and annoyance. The tone of the weight feedback letter largely emphasising long term health risks and sending the parent and child back into the health system for ‘treatment’ seems inappropriate to many parents as this and other studies have now shown [34, 35]. It might therefore be better for the feedback letter to adopt a more neutral tone, alerting parents to their child’s weight status, but also providing parents/guardians with information recognising the complex nature of the environment in which children live and suggesting to parents the best ways of supporting their children to adopt a healthy weight in a simple tone. Local areas should continue to be allowed to modify their letters to try and tailor them more to the needs of their population.

### Implications for research

The current study has shown that just after receiving weight feedback families indicate being more aware about weight issues and resolve to change lifestyles towards healthy eating and more physical activity, however it is not clear what they actually do months after receiving the child weight feedback. There is therefore need for robust longitudinal studies to follow parents/guardians and their children for a significant amount of time well after receiving the weight feedback to identify what sort of behavioural changes families undergo as a result of this feedback. Also further research would be needed to understand the impact of the local initiatives in tackling childhood obesity.

## Conclusions

The current study has indicated clearly that there are potential negative outcomes from the measurement process (emotional distress amongst parents, rejection of the exercise because of fears that children will be over-sensitised about bodies/weight, potential stigmatisation amongst peers), yet there is a strong argument for alerting parents to the dangers of excess weight in children in terms of their future health and clear indications that many struggle to recognise overweight status naturally in an era when overweight is the ‘new normal’.

As the NCMP is now an embedded programme into the future as a national surveillance exercise, and if it is to serve the dual function of helping to trigger behaviour change in parents and children, more careful thought needs to be put into mechanism and process. Health authorities need to pay heed to the evidence about the cycle of emotional reaction of parents to the feedback exercise and to consider when further support could sensibly be brought in. Consideration needs to be given to the re-wording of the feedback letter so that it comes across in a neutral tone, avoiding placing the blame on individuals, acknowledging the influence of the environment surrounding families and aiming to bring families on board to support interventions aimed at combating child weight problems. Local authorities need to think about whether portraying the issue of child weight status as a medical problem and directing them towards GP services is the most sensible direction of travel. Evidence from this and other studies is that parents/guardians and children see child weight status much more as a social issue. Local authorities could therefore use their position to modify the broader environment – removing the health label and providing more holistic approaches to improving the weight status of their populations.

## Additional file

Additional file 1: Interview Schedule for parents/guardians. (DOCX 17 kb)

## Abbreviations

BMI: Body mass index
GP: General practitioner
NCMP: National Child Measurement Programme
PCT: Primary care trust
PSA: Public service agreement
SES: Social economic statius

## Footnotes

1 Responsibility for local public health moved from the primary care trusts to local authorities in 2013.
