# Supplementary material for: Parental response to a letter reporting child overweight measured as part of a routine national programme in England: results from interviews with parents
Source: BMC Public Health. 2016 Aug 20;16:846. doi: 10.1186/s12889-016-3481-3 (PMC4992560; doi:10.1186/s12889-016-3481-3)
Supplement: Additional file 1: — Interview Schedule for parents/guardians. (DOCX 17 kb) [file 12889_2016_3481_MOESM1_ESM.docx]

Exploring Knowledge of parents about the measurement process

- Why do you think children are being weighed and measured at school?
- Who do you think is responsible for this whole process of weighing and measuring children?
- Do you find it legitimate to weigh and measure children at school?
- Do you think it is being handled very well?
- Is it something you would want to be done to your child at school? Why?
- Is it something you would want to be done to all children at school? Why?
- Would you want to talk to your child about weight related issues?
- Why do you think your child has the weight they have?

Exploring Experiences just before child participation in the measurement process

- Do you remember receiving a letter from school asking you to give permission to your child to take part in the measurement process?
- What was your reaction about this letter?
- What did you discuss about this letter with your child?
- What were the feelings of your child towards measurement?
- In your opinion is this likely to be the same reaction for all the children?
- What were the reactions and comments of the other family members?

Exploring experiences of parents after child participation in the measurement process

- What did your child say to you after being measured?
- What was the perception of your child towards the process of measurement?
- Did participating in the measurement process change the curiosity of your child towards his/her weight and height?
- Were there any changes in your and/or your child’s behaviours after participating in the measurement process?
- What were your feelings during the long wait for the measurement results?
- Were you and/or your child eagerly waiting for the measurement results?

Exploring general perceptions and feelings of parents

- How do you feel about your child’s body size at the moment?
- What are some of the reasons to explain your child’s weight status at the moment?
- Do you find it quite easy to tell if your child is about the right weight, underweight or overweight?
- Do you find it easy to speak to your child about weight related issues?
- Are you generally happy about your child?
- Do you feel about your child’s weight, has it changed since they were young?
- How do you feel about your child’s exercise and eating habits?

Exploring experiences of parents during the feedback process

- How did you receive the results from the measurement process?
- How did you feel just before receiving the results of the measurement process?
- How did you feel just after receiving the results of the measurement process?
- How did you deliver these results to your child?
- What was your child’s reaction after receiving these results?
- What were the comments of the other members of the family?

Exploring experiences after the feedback process

- Are there any feelings of worry among the family members due to the results of the measurement process?
- Are there any changes in the way of life of the child due to the results?
- Have you sought some help somewhere about your child’s weight status?
- Who are the providers of help that have been consulted?
- Which help would you recommend to others and why?
- What next is planned about the child’s weight status?

Feelings about lifestyles

- Do you think this measurement process can act as an important spur to families to think of healthy lifestyles?
- Do you think it is important to feedback the height and weight measurement of children to parents and their children?
- Generally what do you make of the whole process?
